# Predictive Modelling and Multi-Objective Optimization of Surface Integrity Parameters in Sustainable Machining Processes of Magnesium Alloy

**DOI:** 10.3390/ma14133547

**Published:** 2021-06-25

**Authors:** Mohd Danish, Saeed Rubaiee, Hassan Ijaz

**Affiliations:** 1 Department of Mechanical and Materials Engineering, University of Jeddah, Jeddah 21589, Saudi Arabia; salrubaiee@uj.edu.sa (S.R.); heagaaz@uj.edu.sa (H.I.); 2 Department of Industrial and Systems Engineering, University of Jeddah, Jeddah 21589, Saudi Arabia

**Keywords:** cryogenic turning, predictive modelling, multi-objective optimization, magnesium alloy, surface integrity, productivity

## Abstract

Magnesium alloys are widely used in numerous engineering applications owing to their superior structural characteristics. However, the machining of magnesium alloy is challenging because of its poor machinability characteristics. Therefore, this paper investigates the machining of magnesium alloys under different sustainable cooling conditions. The machining was performed by varying cutting velocity, feed rate, and depth of cut under dry and cryogenic cooling conditions. The primary focus of the paper is to develop a predictive model for surface roughness under different machining environments. The models developed were found to be in excellent agreement with experimental results, with only 0.3 to 1.6% error. Multi-objective optimization were also performed so that the best surface finish together with high material removal rate could be achieved. Furthermore, the various parameters of surface integrity (i.e., surface roughness, micro-hardness, micro-structures, crystallite size, and lattice strain) were also investigated.

## 1. Introduction

The global environmental problem due to the high consumption of natural resources and the harmful emission of gases has put pressure on manufacturers to use energy-efficient, lightweight materials and sustainable machining techniques [1,2]. Magnesium alloys in particular, having a high strength to weight ratio, are becoming a very attractive choice for the manufacturing industries, especially in the transport sector (automobiles and aviation) [3,4,5]. Since they are lightweight, less energy will be spent, and the harmful emission of gases can be minimized. Moreover, its biocompatibility with the human body and ability to degrade within the body without any known side effects have made it an eminent material for the temporary fixation of implants, where the extra surgery to take out the implant after healing is eliminated, thereby reducing risk, pain and cost [6].

However, its widespread applications are hindered due to two main reasons. Firstly, the high corrosion rate of magnesium alloys in different environments leads to the failure of the component, which hinders their use in widespread applications [7]. Secondly, magnesium alloy, which generally possesses very good machinability (low cutting forces, less power consumption, very low tool wear, and high surface finish), is always associated with ignition risk due to its ability to ignite at low temperatures, and even in the absence of oxygen [8,9]. Its reactivity with water and other oil-based lubricants and coolants makes it a material that is difficult to machine. Hence, near-dry and dry machining of magnesium alloy is considered to be a sustainable machining process [10].

In addition to the dry or near-dry machining, cryogenic machining could be the most suitable sustainable process for machining light alloys [11,12,13]. Using liquefied gases as a coolant, cryogenic machining diminishes the undesirable rise in temperature during machining, as well as eliminating the perilous gases which are associated with other coolants and lubricants [14,15]. The liquefied gas, normally liquid nitrogen (LN2), directly evaporates into the atmosphere immediately after the process, leaving no residue behind and making the process clean and economical [16,17,18]. In addition, the beneficial attribute of lowering the cutting temperature can significantly decrease the ignition risk associated with magnesium alloy [19]. Moreover, cryogenic machining may also help to increase the favorable surface integrity of the machined parts [20,21,22].

In general, failure of a product usually starts from the surface, irrespective of the cause (such as corrosion, crack propagation, or wear) [23]. Surface roughness also influences the bonding strength of the molds used to manufacture medical devices [24]. Hence, the functional performance of each component prepared by any material removal process, particularly machining operation, heavily depends on the quality of the surface produced, including surface topography and subsurface conditions [25]. Among the many surface integrity factors, surface finish (or surface waviness), micro-structure, micro-hardness, and crystallographic planes were reported to be the most significant in terms of assessing the product’s functional quality. Due to the ease in quantification and nondestructive measurement, surface roughness is the most heavily utilized parameter for the assessment of product quality, especially for those products which have gone through metal-removal processes (such as machining). Surface roughness, in addition to the dimensional tolerance of the machined components, also has a major role in the corrosion behaviors of the components.

Similarly, the corrosion rate in magnesium alloys also increases with poor surface finish [26]. Walter et al. (2011) demonstrated that the increased surface roughness tends to cause pitting corrosion in AZ91 magnesium alloy [27]. Uddin et al. (2016) predicted the effects of various machining parameters on the surface roughness, micro-hardness and corrosion resistance while milling biodegradable magnesium alloys. It was reported in their study that lower surface roughness, higher micro-hardness samples were found to have high corrosion resistance [28]. This implies that the comprehensive inspection of the surface roughness of any machined component, especially for magnesium alloy, is critical. Many studies have been done on the surface roughness produced by cryogenically machined parts for titanium-based alloy, Inconel, and steel. However, the studies on surface roughness for cryogenically machined magnesium-based alloys are very few.

A comparison of some current and major studies conducted in recent years on the micro-hardness and surface roughness evaluations for cryogenically machined magnesium alloys is listed in Table 1. Pu et al. (2012) were the first researchers to study the effect of cryogenic conditions on surface roughness, during the orthogonal cutting of AZ31B magnesium alloy [29]. They evaluated the surface quality of the machined parts by studying the arithmetic average of the surface roughness parameter ‘Ra’. The authors found that the surface roughness was reduced by 20% under cryogenic conditions when compared with dry machining for tool nose radii of 30 μm and 70 μm. Dinesh et al. (2015) performed turning operations on ZK60 magnesium alloy under cryogenic and dry conditions and reported that the Ra of cryogenically machined samples was significantly reduced (by about 25–40%) when compared with dry conditions [30]. They also observed that for both cryogenic and dry conditions, an increase in cutting velocity led to better surface finish. Dinesh et al. (2017) further investigated the same alloy under dry and cryogenic conditions for orthogonal cutting operations and found similar enhancement in the surface finish on the machined sample under cryogenic conditions [31]. Danish et al. (2017) also investigated the surface roughness while machining AZ31 magnesium alloy and observed about 56% reduction in average surface roughness (Ra) for cryogenically machined samples compared to dry-machined samples [19].

However, Ra alone does not provide all the details about the surface defects, which can be in the form of high peaks or very low valleys. As discussed above, the corrosion resistance of magnesium alloy is greatly influenced by the surface roughness. Hence, other parameters like the maximum height of profile (Rz) and the total height of profile (Rt) should also be investigated, which is scarcely found in the literature. A combined scheme that incorporates different roughness parameters (Ra, Rz, Rt) and micro-hardness for the magnesium alloy can be very helpful for the manufacturing industries. Material removal rate (MMR) is another important parameter that effectively influences the productivity of the manufacturing system; this is also considered in the present study. Selection of different machining parameters greatly affects the surface roughness, micro-hardness and material removal rate. The selection of optimal machining parameters leads to the combination of minimum surface roughness, maximum micro-hardness, and maximum material removal rate, which could be useful for manufacturing industries and research communities.

As evident from the literature, a comprehensive analysis of surface roughness is needed, especially for magnesium alloys machined under cryogenic conditions. In the present study, a comprehensive analysis of surface roughness was performed for two sustainable machining process (dry and cryogenic) for AZ31 magnesium alloy. Micro-hardness is one of the main parameters by which the functional quality of the component can be assessed, which was also investigated for both machining conditions. Furthermore, mathematical models were developed for predicting different surface roughness and micro-hardness values of the machined samples. Micro-structure, crystallite size and crystalline strain were also investigated. Finally, a multi-objective optimization was also conducted, so that optimal machining parameters were identified where targets of minimum surface roughness, maximum micro-hardness and material removal rate could be achieved.

## 2. Materials and Methods

### 2.1. Work Material, Tool, and Experimental Setup

In the present study, AZ31C Mg alloy was utilized as a work material. The chemical composition of the AZ31C Mg alloy is given in Table 2. Mg alloy rods with a length of 125 mm a diameter of 25 mm were used in the experiments. A carbide cutting tool (ISO NC 3020 CNMG, KORLOY, Seoul, South Korea ) was used for all the machining operations [19,30]. For each experiment, a new tool was used to eliminate any external effect of the used tool on the work material.

**Figure 1:**
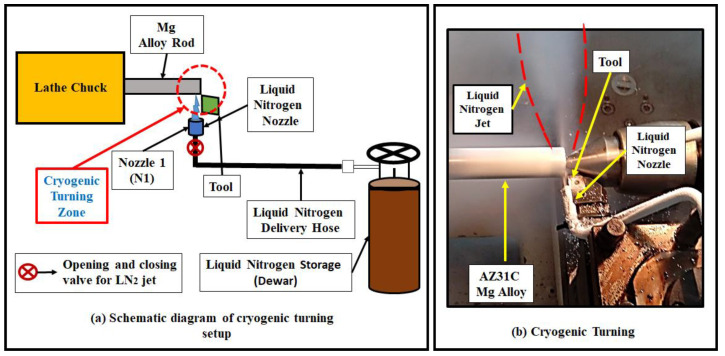
Details of the experimental setup.

Turning experiments were performed on an XL 510 heavy duty lathe. For each experiment, the stroke length was 100 mm. The turning of AZ31C Mg alloy was done considering both dry and cryogenic conditions. For cryogenic turning, a liquid nitrogen delivery extension was attached to the lathe (Figure 1). A liquid nitrogen jet was directed towards the machining zone by adjusting the position of the nozzles at the flank side of the cutting tool (Figure 1a). The liquid nitrogen jet spontaneously evaporated into the environment when it touched the relatively hot surface (work material) during the turning operation, leaving no residue behind and making the process clean (Figure 1b). A K-type thermocouple was used to measure the liquid nitrogen temperature at the nozzle exit. For all the cryogenic turning experiments, an average value of −175 °C was recorded for the liquid nitrogen at the nozzle exit.

### 2.2. Surface Roughness Measurement

**Figure 2:**
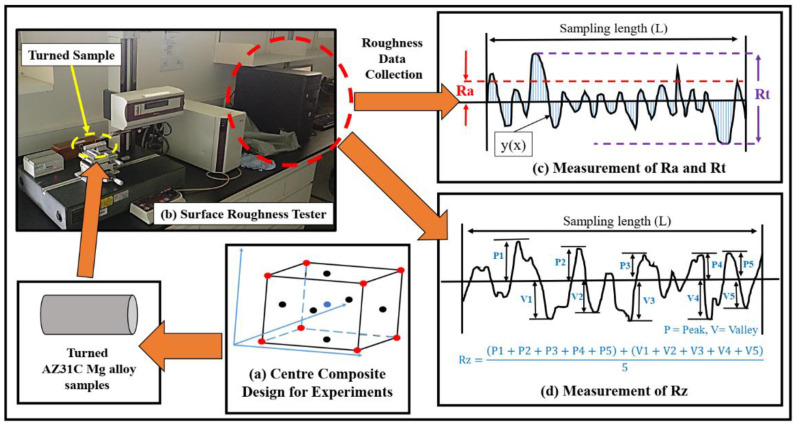
An overview of the surface roughness analysis approach and surface roughness parameters utilized in the study.

The machining of AZ31C Mg alloy under dry and cryogenic conditions was carefully examined for surface roughness using a SV3000 Mitutoyo Surface Roughness tester (Kawasaki, Japan) (Figure 2b). The sampling length of each surface roughness measurement was 10 mm, and each sample was measured five times at five different positions in order to minimize any external effect that could alter the readings. The average of all five readings was taken for further analysis.

As introduced above, three different roughness parameters (i.e., Ra, Rz and Rt) were examined to assess the surface roughness of the machined parts under dry and cryogenic conditions.

An arithmetic average measurement of the roughness parameter (Ra) was done for ISO 4287 [34]. The representation for the calculation of Ra is shown in Figure 2c. According to the definition, Ra can be represented by Equation (1) [35].

(1) $$\mathrm{Ra}=\frac{1}{L}\int_{0}^{L}y(x)dx$$

In the above equation, L and y(x) represent the total measured sample length and the distance between peak and valley, respectively.

The average maximum height of roughness (Rz) can be defined as the arithmetic average of the five successive absolute values of the tallest peaks and lowest valleys on the ISO 4287 sample (Figure 2d) [9]. The average maximum height of roughness can be represented by the Equation (2), which is given below:

(2) $$Rz=\frac{\left|P1\right|+\left|P2\right|+\left|P3\right|+\left|P4\right|+\left|P5\right|+\left|V1\right|+\left|V2\right|+\left|V3\right|+\left|V4\right|+\left|V5\right|}{5}$$

The maximum roughness height (Rt) is the sum of the absolute values of the highest peak and lowest value, as represented in Figure 2c.

### 2.3. Micro-Hardness and Material Removal Rate Measurement

Samples were collected for each set of turning parameters and turning conditions (cryogenic and dry). Micro-hardness (μH) of the machined parts was measured by conducting Vicker’s tests using the “Leco Micro-hardness Tester LM-700AT” machine (St. Joseph, MI, USA). Tests were performed for dwelling time and loading of 10 s and 500 g, respectively. All the measurements for micro-hardness were done according to standard ASTM E384. The micro-hardness of the sample was measured starting from just below the turned surface to the depth where the micro-hardness became constant. Five trials of micro-hardness were performed on each sample to get the average response.

Material removal rate (MMR) may be defined as the volume removed per unit time and effectively depends on three machining parameters: cutting velocity, feed rate and depth of cut. The following equation can be used to calculate MRR [36].

MRR = Vc · f · d(3)

In the above equation, Vc, f and d represent the cutting velocity, feed rate and depth of cut, respectively. A high MRR value results in low machining time, which is correlates with a high production rate. However, a high MRR also affects the surface quality. Hence, it is important to determine the optimal MRR value without compromising surface quality.

### 2.4. Design of Experiment

Experiments were conducted considering three different machining parameters, namely depth of cut (d), feed rate (f) and cutting velocity (Vc), to investigate their effects on the surface roughness of the recently machined part. Due to the low melting point of magnesium alloy, ignition risk can be reduced by selecting lower range machining parameters [37,38].

Surface roughness of the machined part can be influenced by multi-variable inputs, such as depth of cut, feed rate and cutting velocity [39,40,41]. Response surface methodology (RSM) is a useful tool for developing empirical relations between multi-variable and multi-response systems [42]. In the present study, different empirical models for various surface roughness parameters (Ra, Rz and Rt) and micro-hardness were developed by employing RSM. Rotatable face-centered center composite design (FC-CCD) experimental methodology was adopted, which has six center points: one point at each corner and each face, as shown in Figure 2a. The turning parameters, together with their respective ranges and responses, are given in Table 3. Each turning process was done twice to ensure the repeatability of the experiments.

After conducting all the experiments and compiling the data, RSM was employed for further analysis. It was used to model the relationship between the various surface roughness responses and micro-hardness with the turning parameters (Vc, f and d), as shown by Equation (4).

(4) Y=f(Vc,f,d)+e

For developing these models, a full quadratic polynomial with nine degrees of freedom was used. It includes linear, quadratic and interaction terms, represented by Equation (5).

(5) $$Y=\beta_{0}+\sum_{i=1}^{p}\beta_{i}X_{i}+\sum_{i=1}^{p}\beta_{ii}X_{i}^{2}+\sum_{i=1}^{p-1}\sum_{j=1}^{p}\beta_{ij}X_{i}X_{j}$$

Regression analysis was implemented on the responses obtained after conducting the experiments, in er to determine the relation. Statistical techniques such analysis of variance (ANOVA) and the F test were applied to assess the significance of the whole model and its inherent individual terms. A particular term and whole model were considered significant only if the *p*-value was smaller than 0.05 [42]. The adequacy of the model was also checked against normal probability plots. Five different experiments with five random sets of input variables (Vc, f and d) for each condition were performed to verify the reliability of the model.

## 3. Results and Discussion

The full experimental scheme with all the input variable parameters and all the responses for each turning condition conducted according to FC-CCD is given in Table 4.

### 3.1. Statistical Analysis and Empirical Modelling

Analysis of variance (ANOVA) was used to study the effects of input parameters on output responses.

The models developed for the surface roughness assessment of dry machined samples are given in Table 5. All the models used to evaluate the surface roughness parameters (Ra, Rz and Rt) showed significance, as they have *p*-values less than 0.05 (Table 5). The individual factor significance can also be shown by the same method. In the case of Ra, all the individual factors demonstrated significance. However, Rz and Rt each have one non-significant factor (Vc^2^ and f·d, respectively), as the *p*-values were found to be greater than 0.05. On the other hand, the R-squared value was found to be very close to 1 for each surface roughness model developed for dry turning, which shows the goodness of fit of data, as mentioned in Table 5. Also, the Adj. R-Squared value was in good agreement with the Pred. R-squared value.

The results for the surface roughness model obtained from ANOVA for the cryogenic turning conditions are given in Table 6. The results for the surface roughness model presented in Table 6 indicated significance, as the *p*-value was greater than 0.05. All the individual factors of the Ra model were found to be significant. However, for Rz, the factors Vc^2^ and f·d had *p*-values of 0.76 and 0.19, respectively, which are both higher than 0.05, and are thus not significant for the model. For Rt, all the factors of the model except for Vc and d were significant. The R-squared value was approaching 1 and the Adj. R-Squared value was close to the Pred. R-squared value for all the surface roughness models (Table 6), which indicates the goodness of fit of the data.

The *p*-values for micro-hardness under both dry and cryogenic conditions were less than 0.05, hence indicating significance (Table 7). Unlike the models developed for surface roughness, many individual factors were found to be insignificant for the micro-hardness models. Three factors (Vc^2^, Vc·f and Vc·d) for dry conditions and four factors (d, f^2^, Vc·d and f·d) for cryogenic conditions were found to be insignificant for the micro-hardness model (Table 7). The R-squared value was 0.996 and 0.988 for the micro-hardness model developed for dry and cryogenic conditions, respectively. As desired, Adj. R-Squared and Pred. R-squared values were in close agreement with each other for both micro-hardness models.

All the developed models (Equation (6) to Equation (13)) of the surface roughness and micro-hardness for the AZ31C magnesium alloy under dry and cryogenic cutting conditions are given below.

(6) $$\mathrm{Ra}=-0.0036\;\mathrm{Vc}-16.096f+0.018d-3.1\times 10^{-6}{\mathrm{Vc}}^{2}+75.795f^{2}-0.279d^{2}+0.011\mathrm{Vcf}+0.003\mathrm{Vcd}\;+1.281\mathrm{fd}+1.769$$

(7) $$\mathrm{Rz}=-0.012\;\mathrm{Vc}-29.724f-1.203d+3.7\times 10^{-5}{\mathrm{Vc}}^{2}+184.204f^{2}+0.242d^{2}-0.066\mathrm{Vcf}+0.002\mathrm{Vcd}\;+11.469\mathrm{fd}+6.803$$

(8) $$\mathrm{Rt}=-0.028\;\mathrm{Vc}-174.294f+2.028d-5.6\times 10^{-5}{\mathrm{Vc}}^{2}+833.182f^{2}-3.481d^{2}+0.094\mathrm{Vcf}+0.028\mathrm{Vcd}\;+3.031\mathrm{fd}+18.289$$

(9) $$\mu H=-0.201\;\mathrm{Vc}-295.409f-5.464d+6.67\times 10^{-4}{\mathrm{Vc}}^{2}+1920.45f^{2}-12.045d^{2}-0.112\mathrm{Vcf}-0.019\mathrm{Vcd}\;+90.625\mathrm{fd}+88.622$$

(10) $$\mathrm{Ra}=-0.002\;\mathrm{Vc}-9.885f+0.257d+4.56\times 10^{-6}{\mathrm{Vc}}^{2}+52.273f^{2}-0.102d^{2}+0.003\mathrm{Vcf}-0.001\mathrm{Vcd}\;+\mathrm{fd}+0.924$$

(11) $$\mathrm{Rz}=-0.04\;\mathrm{Vc}+64.729f+0.189d+9.73\times 10^{-5}{\mathrm{Vc}}^{2}-220.455f^{2}-0.042d^{2}+0.06\mathrm{Vcf}+0.007\mathrm{Vcd}\;-1.125\mathrm{fd}-0.194$$

(12) $$\mathrm{Rt}=-0.013\;\mathrm{Vc}-119.723f-3.12d-3.72\times 10^{-5}{\mathrm{Vc}}^{2}+574.66f^{2}+2.621d^{2}+0.078\mathrm{Vcf}-0.004\mathrm{Vcd}\;+31.812\mathrm{fd}+10.793$$

(13) $$\mu H=0.305\;\mathrm{Vc}+679.227f-80.759d-7.4\times 10^{-4}{\mathrm{Vc}}^{2}-1386.36f^{2}+94.886d^{2}-1.012\mathrm{Vcf}+0.004\mathrm{Vcd}\;+21.875\mathrm{fd}+32.872$$

After developing the models (Equation (6) to Equation (13)) using RSM, the adequacy and efficiency of the models were analyzed. This was done by analyzing the normal plot of residuals and residual verses predicted plots, and by checking the maximum percentage error between the obtained and experimental results.

**Figure 3:**
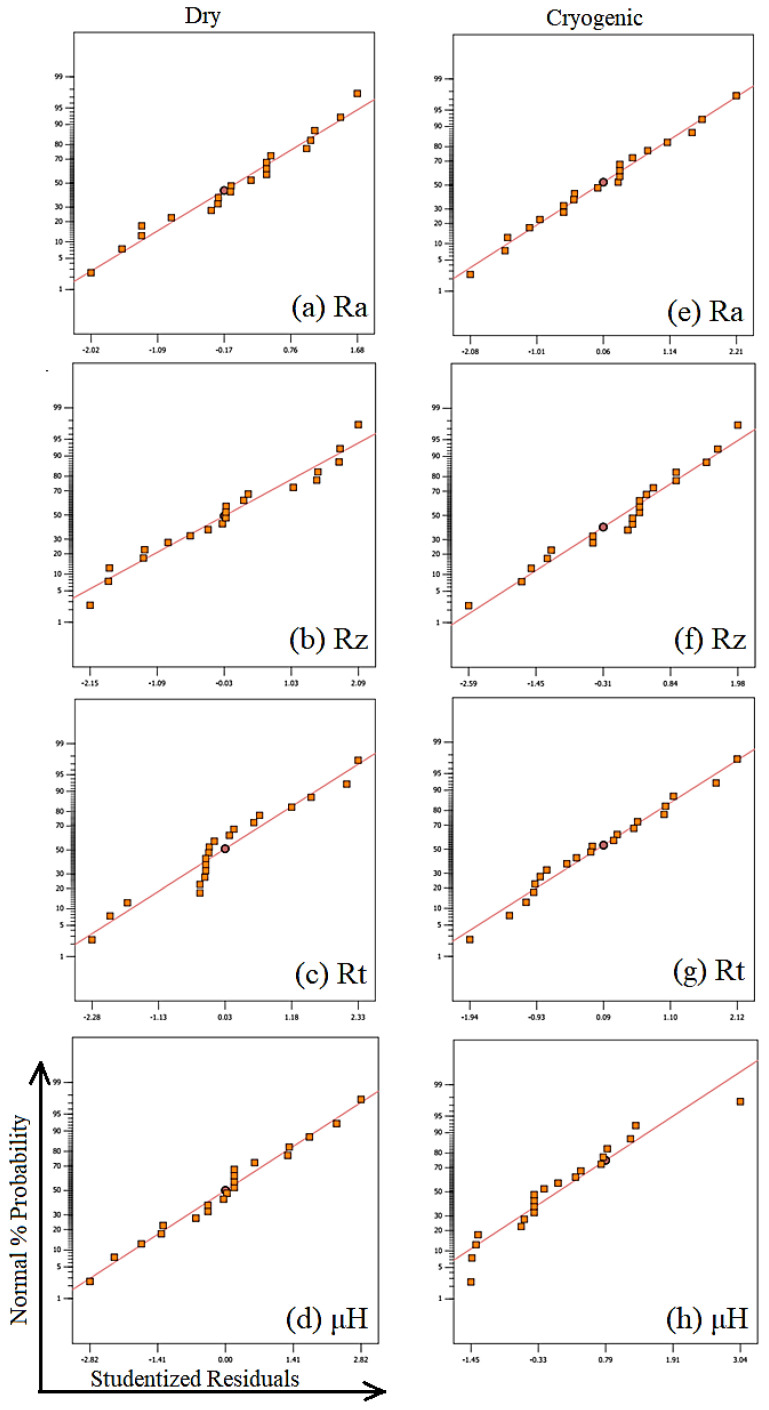
Normal plots of residuals of all the developed models for turning AZ31 Mg alloy.

The normal plot of residuals of all the models is shown in Figure 3. For a model to predict in the desired designed space, the points in the normal plot of residuals should not follow any particular trend [43]. A trend shaped like the letter “S” is highly undesirable. Furthermore, the points should be distributed randomly and should also follow a straight line. This shows that the model does not carry any abnormal behavior. In the present study, all the models showed desirable characteristics in the normal plots of residuals, which is evident from Figure 3. Hence, it can be concluded that the models were working normally, which is desirable for a good model.

**Figure 4:**
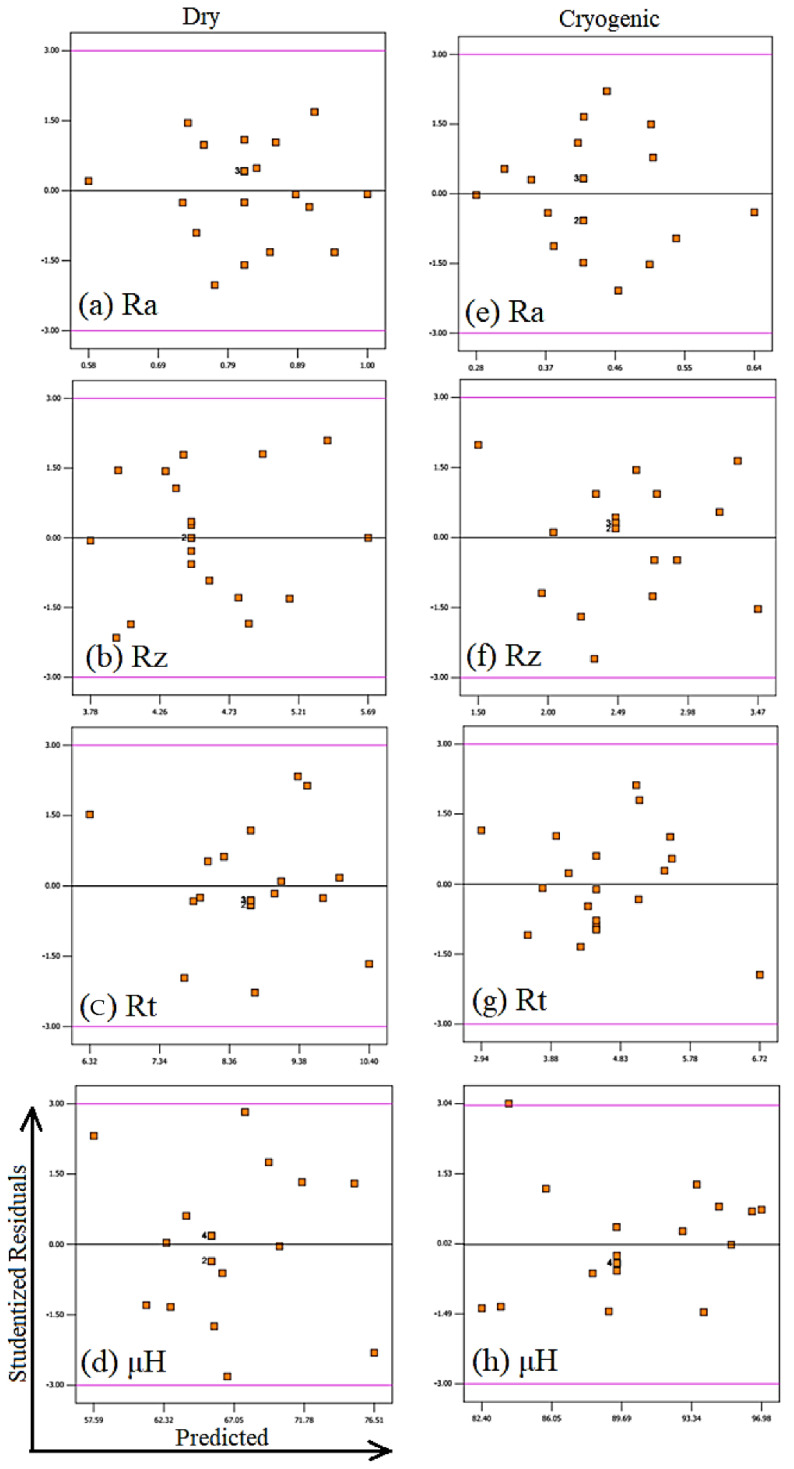
Residuals vs. predicted plots of all the developed models for turning AZ31 Mg alloy.

The models were also checked for any constant error occurred during the experimentations. This can be visualized with residual verses predicted plots. The points in these plots should be randomly distributed and should not exceed the limits (−3 to 3). The residual verses predicted plots of the models developed in the current study were found to be in good agreement with the above statement, as evident in Figure 4. This implies that the models were free from any kind of constant error, which is a desirable.

The efficiency of the model was also checked by evaluating the percentage error (Equation (14)) between the predicted and the experimental values.

(14) $$\mathrm{Percentage}\;\mathrm{error}=\left(\frac{\left(\mathrm{Experimental}\;\mathrm{value}-\mathrm{Predicted}\;\mathrm{value}\right)}{\mathrm{Experiemntal}\;\mathrm{value}}\right)\times 100$$

The maximum percentage errors for all the models are shown in Table 8. As shown in the table, all models had good efficiency, having maximum percentage errors ranging from 0.3 to 1.63%, which is for the model developed for Ra in dry turning and μH in cryogenic turning, respectively.

### 3.2. Influence of Process Parameters

**Figure 5:**
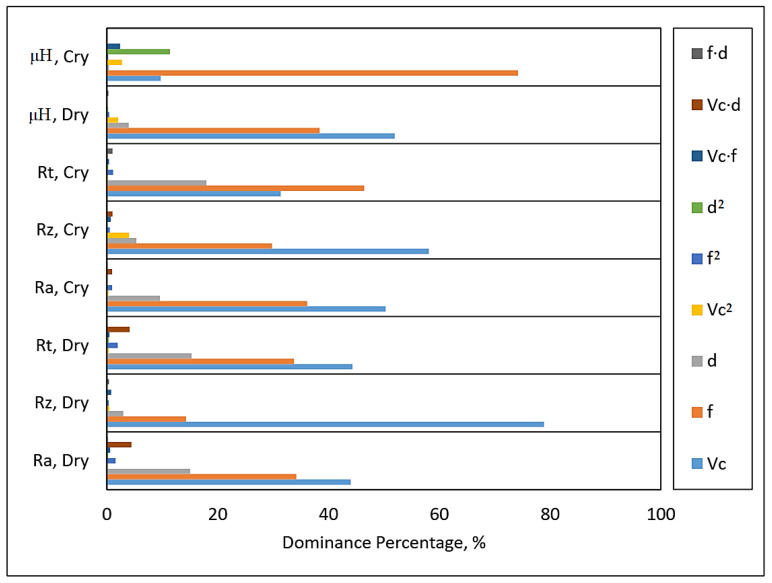
Dominance percentage of each process parameter in different turning conditions.

The influence of different machining parameters (Vc, f and d) on the responses (Ra, Rz, Rt and μH) are discussed in this section. At first, it was important to identify the dominance of the above-mentioned machining parameters on both micro-hardness and surface roughness using the statistical analysis done by ANOVA. The percentage dominance of each selected parameter on micro-hardness and surface roughness is shown in Figure 5.

One can observe from Figure 5 that the surface roughness and micro-hardness were mostly affected by feed rate and cutting velocity. Hence, both feed rate and cutting velocity were identified to perform the parametric analysis.

**Figure 6:**
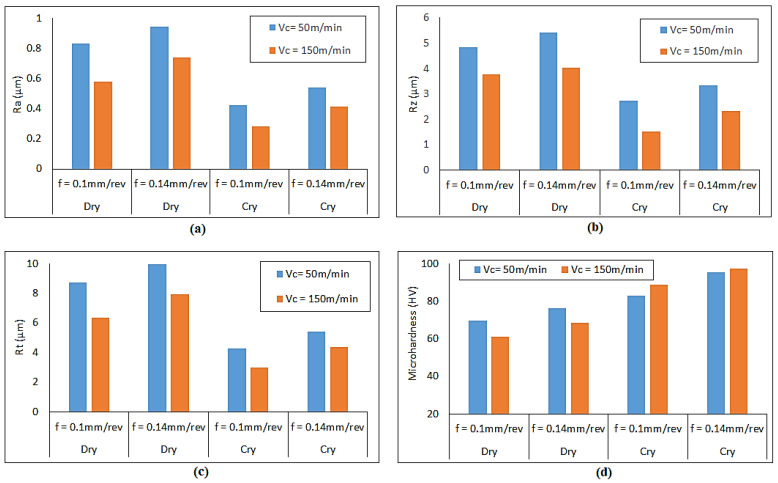
Variation of different responses with cutting parameters and conditions: (**a**) Ra; (**b**) Rz; (**c**) Rt; and (**d**) micro-hardness (μH).

The variations observed in the micro-hardness and surface roughness during the turning operation of magnesium alloy under dry and cryogenic conditions due to feed rate and cutting velocity are shown in Figure 6.

All the surface roughness parameters showed a decreasing trend with an increase in cutting velocity for both dry and cryogenic conditions. On the contrary, surface roughness parameters showed an increasing trend with feed rate. However, the magnitude of surface roughness parameters was significantly reduced under cryogenic conditions. This reduction in the surface roughness may be attributed to the flow of liquid nitrogen (LN2) in the targeted machining zone. As soon as LN2 touches the relatively hot surface during the cutting process, it changes its phase, producing a high volume of gas. This gas provides a cushioning effect during the turning operation and hence dampens vibrations which may occur during the process [44]. Furthermore, the liquid nitrogen also acts like a lubricant during the turning process, thereby decreasing the frictional forces [15]. These two effects are the possible reasons behind the dramatic reduction in the different surface roughness parameters.

Micro-hardness was found to increase after the turning operations. This was to be expected, because turning is associated with severe plastic deformation, during which strain hardening occurs [45,46]. Parametric analysis suggested that the micro-hardness decreased with an increase in cutting velocity for dry turned samples of magnesium alloy, which can be observed in Figure 6d. This decreasing trend can be associated with the thermal softening due to high temperatures in the cutting zone during the dry turning process [29]. On the other hand, micro-hardness increased with feed rate. Under cryogenic conditions, high values of micro-hardness were obtained for higher cutting velocities (Figure 6d). The reason for this behavior could be low temperature values in the cutting zone caused by the liquid nitrogen. The combination of this low temperature during cryogenic turning operation and high cutting velocity and feed rate may result in more dominant dynamic recrystallization, thereby increasing the strain hardening effect [31,44]. Hence, a significant increase in micro-hardness after the cryogenic turning was observed.

**Figure 7:**
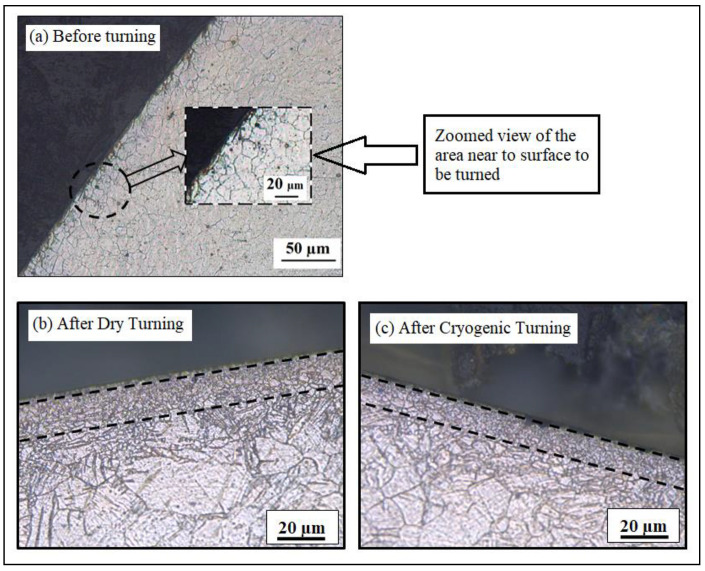
Micro-structures of the samples (**a**) before turning, (**b**) after dry turning and (**c**) after cryogenic turning.

The increase in micro-hardness could also be due the occurrence of grain refinement from the turning operations [47,48]. Accordingly, the micro-structure of the as-received sample, dry-turned sample and cryogenically turned sample were also taken (Figure 7). Before any turning process, the micro-structure near the surface of the sample can be seen in Figure 7a, with an average grain size of approximately 20 μm. The initial micro-hardness value on the surface of the sample before the turning operation was 53 HV.

The micro-structures of the samples after turning operations (dry and cryogenic) at Vc = 150 m/min, f = 0.14 mm/rev and d = 0.2 mm are shown in Figure 7. It can be seen that grain refinement occurred after the turning operation in both environments. Especially near the turned surface, a grain refinement layer was observed, with a very small grain size compared to the other part of the sample. In addition to strain hardening, this grain refinement layer could be the reason for the higher micro-hardness in the samples after turning operations [47].

For the case shown in Figure 7b,c, the micro-hardness was 68.3 HV for dry- and 97.2 HV for cryogenically turned samples. The higher value of micro-hardness in cryogenically turned samples is due to the more dominant dynamic recrystallization that occurred due to low temperatures during the cutting process, which leads to even smaller grain sizes [31].

**Figure 8:**
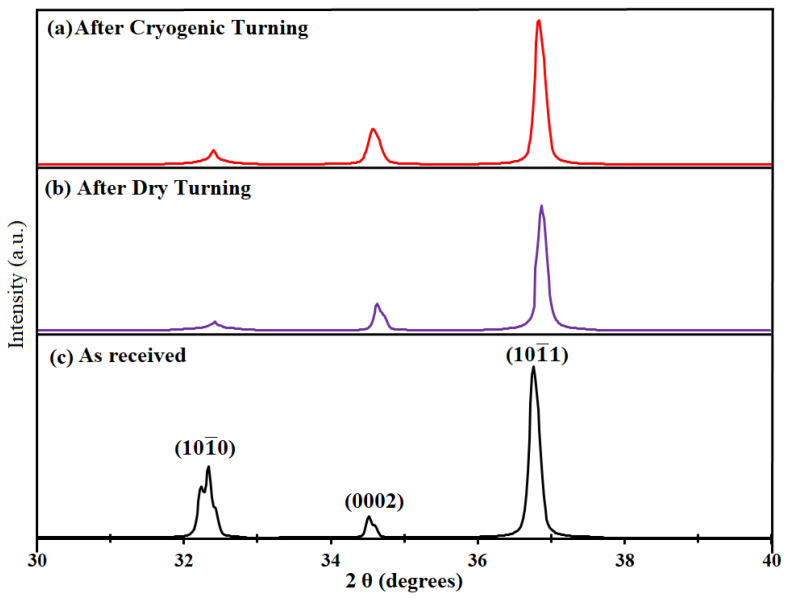
XRD patterns of the AZ31C magnesium alloy samples.

The grain boundaries are not very clear in this zone, due to small grain sizes, as evident from Figure 7. That is why further investigation was needed to examine the micro-hardness behavior. For this reason, X-ray diffraction (XRD) patterns were used. It has been reported that XRD patterns can be used to investigate the strain hardening of materials by analyzing the crystallite size, which is inversely proportional to the peak width [49,50]. The XRD patterns for the same samples which were shown in Figure 7 are shown in Figure 8. The three major peaks associated with the magnesium alloy are shown in the XRD patterns. They represent the basal (0002) and slip planes (($101\bar{0}$) and ($101\bar{1}$)) of the material. The three major peaks (with the highest intensities) were the prime focus for analyzing and studying the lattice strain and crystallite size. The full width at half maximum (FWHM) of each peak was calculated with the help of “Xpert Highscore Plus” software.

**Figure 9:**
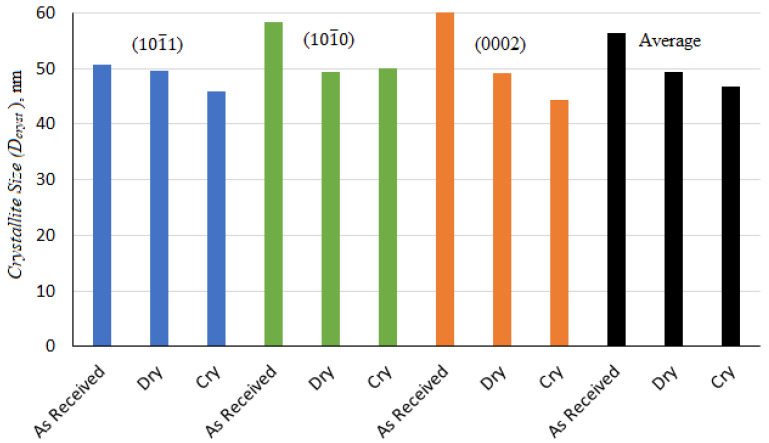
Crystallite size for each peak and the average crystallite size for the as-received sample, after dry turning and after cryogenic turning.

**Figure 10:**
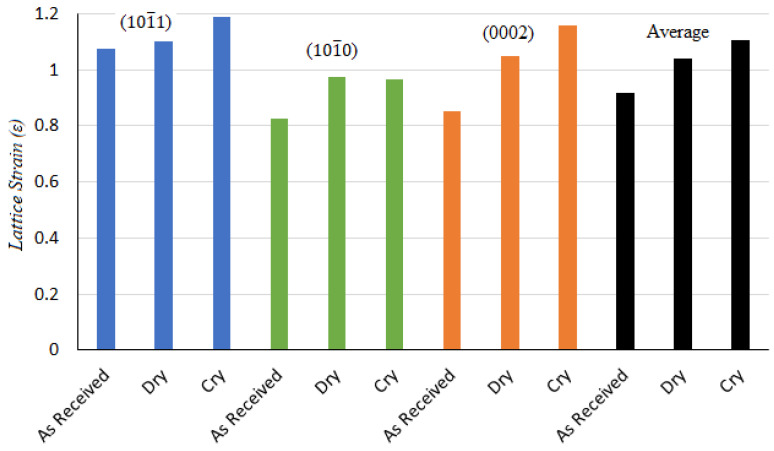
Lattice strain for each peak and the average crystallite size for the as-received sample, after dry turning and after cryogenic turning.

Thereafter, crystallite size and lattice strain were measured by using Equations (15) and (16), respectively [51].

(15) $$W=\frac{0.9\;\lambda }{D_{\mathrm{cryst}}\cos\theta }$$

(16) $$W.\cos\theta =\left(\frac{0.9\;\lambda }{D_{\mathrm{cryst}}}\right)+\left(4\epsilon \sin\theta \right)$$

where W is defined as the full width at half maximum of a peak (FWHM), Dcryst is defined as crystallite size, and ε and λ are the lattice strain and wavelength, respectively. θ represents the Bragg’s angle. The crystallite size and lattice strain for all the major peaks are shown in Figure 9 and Figure 10. As each peak gives their respective crystallite size and lattice strain, an average was also measured to achieve a better understanding.

It is evident from Figure 9 that the crystallite size of the magnesium alloy decreased and was smallest for the samples turned in a cryogenic environment. The average crystallite size for the as-received sample was 56.37 nm. However, the average crystallite size after the dry turning and cryogenic turning processes was found to be about 49.38 nm and 46.7 nm, respectively. This decrease in crystallite size after the turning process was found to agree with the micro-structure results, where refined grain layers could be seen on the machined surfaces of the samples (Figure 7). The crystallite size is inversely proportional to the lattice strain (which is also called work hardening), hence, the exact opposite trend can be seen for lattice strain compared to Dcryst, as shown in Figure 10. Both crystallite size and lattice strain are a function of FWHM, which broadened after the turning operations. The broadening of FWHM was more significant in the case of cryogenically turned magnesium samples, which can be attributed to high plastic deformation and low temperature. The results of lattice strains for the magnesium alloy samples also support the experimental results of the micro-hardness results (Figure 6).

### 3.3. Optimization of Process Parameters

The main objective of this section is to identify the optimal machining parameters that may help to find the minimum surface roughness, maximum micro-hardness and material removal rate (MMR) values [36]. According to the observation in the previous section, cryogenic turning was found to be more beneficial for producing high finish surfaces with higher micro-hardness. That is why cryogenic turning was considered for the optimization.

The desirability approach was considered for the optimization, as it was found to be a powerful technique for coping with multi-objectives and multi-responses [36,52]. Therefore, in the current study, the desirability function (DF) technique was employed to optimize the various machining parameters for different optimizations. All the objectives together with their respective ranges and importance are shown in Table 9.

Here, two main cases were considered for the multi-objective optimization, which are as follows:

- Case 1: Minimize surface roughness parameters (Ra, Rz and Rt) with maximum micro-hardness (μH).
- Case 2: Minimize surface roughness parameters (Ra, Rz and Rt) with maximum micro-hardness (μH) and maximum material removal rate (MMR).

**Figure 11:**
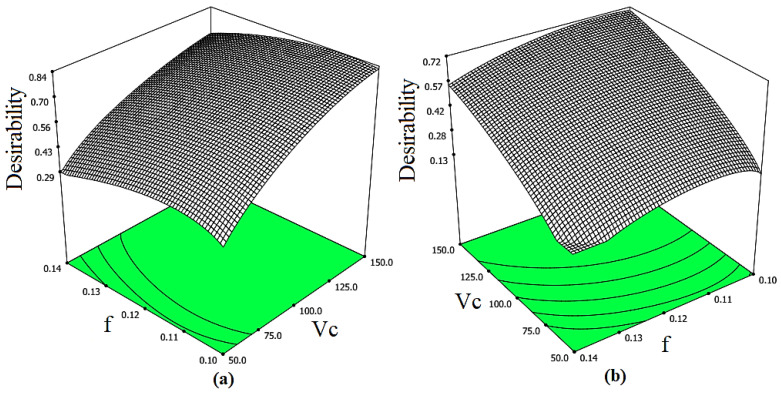
3D Plot of desirability for the optimal solutions of (**a**) Case 1 and (**b**) Case 2.

The 3D plots of desirability of the optimal solutions according to the DF for both cases are shown in Figure 11.

The optimal solution with the maximum desirability was selected. The optimal solution obtained for each case is reported in Table 10.

While comparing the optimal solutions for both cases, it was observed that the difference between the surface roughness parameters and micro-hardness is very small. However, the difference between the MMR of each case is very high and significant. This shows that the optimization of the parameters for surface roughness, micro-hardness and MMR simultaneously gives a more beneficial solution. By utilizing Case 2, enhanced surface quality and higher productivity for the magnesium alloy can be achieved.

## 4. Conclusions

The main conclusions are as follows:

- The mathematical predictive models for surface roughness parameters (Ra, Rz, and Rt) and micro-hardness of the turned AZ31 magnesium alloy samples were successfully developed for both sustainable machining processes. The results predicted by the proposed models were in close agreement with the experimental ones (0.3–1.6%).
- The parametric analysis shows that micro-hardness and surface finish of the machined samples were most affected by the cutting parameters, namely cutting velocity and feed rate. For surface roughness, the most dominant factor was cutting velocity, irrespective of the turning environment. However, for the micro-hardness of the machined sample turned under cryogenic conditions, the most dominant factor was the feed rate.
- Better surface finish of the machined samples was obtained under cryogenic conditions compared to dry conditions. However, surface roughness showed a decreasing trend with the cutting velocity and an increasing trend with the feed rate for both dry and cryogenic cutting conditions.
- Higher micro-hardness was measured for all machined samples. Micro-structural and XRD analysis of the machined samples confirmed this finding. Grain refinement layers were found on the machined samples, due to strain hardening. Moreover, from XRD, it was found that the crystallite size was smaller on the machined samples, which is in good agreement with the micro-structural results. Furthermore, lattice strain was higher in the turned samples and was highest for cryogenic cutting conditions, which is in close agreement with the micro-hardness results.
- Two multi-objective optimization cases were conducted in the present study:
  ➢ In the first case, the objective was to maximize the micro-hardness and minimize the surface roughness. The optimal turning parameters were found to be Vc = 150 m/min, f = 0.11 rev/min, and d = 0.2 mm, with results Ra = 0.292 μm, Rz = 1.707 μm, Rt = 3.065 μm, and micro-hardness = 90.79 HV.
  ➢ For the second case, maximization of MMR was also included in the objective. The optimal result for this case was Vc = 150 m/min, f = 0.11 rev/min, and d = 0.6 mm, with results Ra = 0.332 μm, Rz = 2.153 μm, Rt = 3.793 μm, micro-hardness = 90.8 HV and MMR = 9.9 mm^3^/min.

## Figures and Tables

**Table 1:** Studies conducted on surface roughness and micro-hardness of magnesium alloys machined in a cryogenic environment.

Author/Year [Reference]	Material Removal Process	Work Material	Surface Roughness Parameters			Micro-Hardness
			Ra	Rz	Rt	
Pu et al. (2012) [29]	Burnishing	AZ31B- Mg	√	-	-	-
Kheireddine et al. (2013) [28]	Drilling	AZ31B-Mg	-	-	-	√
Dinesh et al. (2015) [30]	Turning	ZK60 Mg	√	-	-	√
Dinesh et al. (2017) [31]	Orthogonal cutting	ZK60 Mg	√	-	-	√
Danish et al. (2017) [19]	Turning	AZ31 Mg	√	-	-	-
Shen et al. (2017) [32]	Orthogonal cutting	AZ31B Mg	-	-	-	√
Danish et al. (2019) [33]	Turning	AZ31C Mg	√	-	-	√
CURRENT STUDY	Turning	AZ31 Mg	√	√	√	√

**Table 2:** Chemical composition of AZ31C mg alloy.

Element	Aluminum	Zinc	Manganese	Mg
Weight %	3.42	0.97	0.14	Balance

**Table 3:** Turning parameters, cooling techniques and response parameters.

Turning Parameter and Levels			Cooling Techniques	Responses		
Cutting velocity (Vc), m/min	Feed rate (f), mm/rev	Depth of cut (d), mm	1. Dry 2. Cryogenic turning with LN_2_ jet	Surface roughness Ra (μm) Rz (μm) Rt (μm)	Micro-hardness μH, (Hv)	Material Removal rate MRR, (mm^3^/min)
50	0.10	0.2				
100	0.12	0.4				
150	0.14	0.6				

**Table 4:** Turning parameters, cooling techniques and response parameters.

S. no.	Vc	f	d	Dry Turning				Cryogenic Turning				MRR.
				Ra	Rz	Rt	μH	Ra	Rz	Rt	μH	
1	150.00	0.10	0.20	0.582	3.781	6.338	60.9	0.281	1.513	2.967	88.6	3
2	100.00	0.12	0.40	0.815	4.473	8.659	65.4	0.421	2.472	4.495	89.1	4.8
3	100.00	0.12	0.40	0.815	4.473	8.662	65.6	0.421	2.47	4.456	89.2	4.8
4	150.00	0.14	0.20	0.742	4.046	7.927	68.3	0.414	2.334	4.376	97.2	4.2
5	100.00	0.12	0.20	0.755	4.38	8.056	66.1	0.381	2.303	4.133	94.2	2.4
6	100.00	0.10	0.40	0.768	4.312	8.293	62.5	0.374	2.033	3.996	85.2	4
7	100.00	0.14	0.40	0.921	4.783	9.724	70.1	0.51	2.72	5.543	93.3	5.6
8	150.00	0.12	0.40	0.722	3.987	7.656	62.4	0.353	2.215	3.768	89.6	7.2
9	100.00	0.12	0.60	0.851	4.587	9.016	64	0.453	2.623	5.156	93	7.2
10	100.00	0.12	0.40	0.816	4.477	8.662	65.4	0.419	2.471	4.461	89.2	4.8
11	100.00	0.12	0.40	0.814	4.469	8.659	65.6	0.42	2.471	4.451	89.2	4.8
12	50.00	0.10	0.60	0.862	4.976	9.118	66.1	0.506	2.898	5.067	82	3
13	150.00	0.10	0.60	0.731	3.944	7.829	58	0.318	1.946	3.543	88	9
14	100.00	0.12	0.40	0.812	4.478	8.662	65.6	0.42	2.47	4.531	89.2	4.8
15	50.00	0.12	0.40	0.891	5.134	9.414	72	0.512	3.201	5.125	86.3	2.4
16	50.00	0.14	0.20	0.948	5.422	9.973	76.1	0.541	3.331	5.434	95.4	1.4
17	100.00	0.12	0.40	0.815	4.465	8.706	65.6	0.421	2.471	4.452	89.3	4.8
18	150.00	0.14	0.60	0.911	4.432	9.527	65.4	0.465	2.762	5.541	96.7	12.6
19	50.00	0.14	0.60	0.998	5.687	10.38	75.4	0.643	3.46	6.674	95	4.2
20	50.00	0.10	0.20	0.833	4.855	8.699	69.7	0.422	2.738	4.256	83	1

Vc–cutting velocity (m/min), f–feed rate (mm/rev), d–depth of cut (mm), Ra–arithmetic average roughness (μm), Rz–average maximum height of roughness (μm), Rt–maximum roughness height (μm), μH–micro-hardness (HV) and MMR–material removal rate (mm^3^/min).

**Table 5:** ANOVA table for surface roughness for dry turning.

Dry Turning	Source	SOS	DOF	Mean Square	F-Value	*p*-Value
Ra	Model	0.161832566	9	0.017981396	7125.834759	<0.0001
	Vc	0.0712336	1	0.0712336	28229.11285	<0.0001
	f	0.0553536	1	0.0553536	21936.03891	<0.0001
	d	0.0243049	1	0.0243049	9631.771593	<0.0001
	Vc^2^	0.000162278	1	0.000162278	64.30919571	<0.0001
	f^2^	0.002527778	1	0.002527778	1001.731514	<0.0001
	d^2^	0.000343841	1	0.000343841	136.2604701	<0.0001
	Vc·f	0.000990125	1	0.000990125	392.3759344	<0.0001
	Vc·d	0.007140125	1	0.007140125	2829.555075	<0.0001
	f·d	0.000210125	1	0.000210125	83.27028731	<0.0001
	Residual	13.552	10	1.355	-	-
	R-Squared = 0.9998, Adj R-Squared = 0.9997 and Pred R-Squared = 0.9992					
Rz	Model	4.385992	9	0.487332	2131.035	<0.0001
	Vc	3.462146	1	3.462146	15139.47	<0.0001
	f	0.626	1	0.626	2737.411	<0.0001
	d	0.130416	1	0.130416	570.2924	<0.0001
	Vc^2^	0.020663	1	0.020663	90.35539	<0.0001
	f^2^	0.01493	1	0.01493	65.2858	<0.0001
	d^2^	0.000258	1	0.000258	1.127228	0.3133
	Vc·f	0.034453	1	0.034453	150.6586	<0.0001
	Vc·d	0.003321	1	0.003321	14.52281	0.0034
	f·d	0.016836	1	0.016836	73.62198	<0.0001
	Residual	0.002287	10	0.000229	-	-
	R-Squared = 0.9995, Adj R-Squared = 0.9990 and Pred R-Squared = 0.9934					
Rt	Model	15.56307	9	1.72923	1742.162	<0.0001
	Vc	6.900625	1	6.900625	6952.231	<0.0001
	f	5.262052	1	5.262052	5301.404	<0.0001
	d	2.378513	1	2.378513	2396.301	<0.0001
	Vc^2^	0.054075	1	0.054075	54.47954	<0.0001
	f^2^	0.305444	1	0.305444	307.7287	<0.0001
	d^2^	0.053307	1	0.053307	53.70529	<0.0001
	Vc·f	0.0705	1	0.0705	71.02736	<0.0001
	Vc·d	0.641278	1	0.641278	646.0739	<0.0001
	f·d	0.001176	1	0.001176	1.184921	0.3019
	Residual	0.009926	10	0.000993	-	-
	R-Squared = 0.9993, Adj R-Squared = 0.9987 and Pred R-Squared = 0.9940					

**Table 6:** ANOVA table for surface roughness for cryogenic turning.

Cryogenic Turning	Source	Sum of Squares	Degree of Freedom	Mean Square	F-Value	*p*-Value
Ra	Model	0.124974	9	0.013886	10042.47	<0.0001
	Vc	0.062885	1	0.062885	45478.89	<0.0001
	f	0.045158	1	0.045158	32658.93	<0.0001
	d	0.011972	1	0.011972	8657.962	<0.0001
	Vc^2^	0.000358	1	0.000358	258.8798	<0.0001
	f^2^	0.001202	1	0.001202	869.4938	<0.0001
	d^2^	4.6 × 10^−5^	1	4.6 × 10^−5^	33.28402	0.0002
	Vc·f	7.2 × 10^−5^	1	7.2 × 10^−5^	52.07101	<0.0001
	Vc·d	0.001201	1	0.001201	868.2117	<0.0001
	f·d	0.000128	1	0.000128	92.57068	<0.0001
	Residual	1.38 × 10^−5^	10	1.38 × 10^−6^	-	-
	R-Squared = 0.9998, Adj R-Squared = 0.9998 and Pred R-Squared = 0.9991					
Rz	Model	4.061161	9	0.45124	5552.296	<0.0001
	Vc	2.360016	1	2.360016	29038.88	<0.0001
	f	1.210344	1	1.210344	14892.71	<0.0001
	d	0.21609	1	0.21609	2658.885	<0.0001
	Vc^2^	0.16281	1	0.16281	2003.303	<0.0001
	f^2^	0.021384	1	0.021384	263.1211	<0.0001
	d^2^	7.78 × 10^−6^	1	7.78 × 10^−6^	0.09571	0.7634
	Vc·f	0.029041	1	0.029041	357.3296	<0.0001
	Vc·d	0.040898	1	0.040898	503.2305	<0.0001
	f·d	0.000162	1	0.000162	1.993333	0.1883
	Residual	0.000813	10	8.13 × 10^−5^	-	-
	R-Squared = 0.9998, Adj R-Squared = 0.9996 and Pred R-Squared = 0.9982					
Rt	Model	12.86389	9	1.429322	489.277	<0.0001
	Vc	4.046232	1	4.046232	1385.082	<0.0001
	f	5.989212	1	5.989212	2050.192	<0.0001
	d	2.318423	1	2.318423	793.6287	<0.0001
	Vc^2^	0.023855	1	0.023855	8.165749	0.0170
	f^2^	0.145303	1	0.145303	49.73911	<0.0001
	d^2^	0.03024	1	0.03024	10.3516	0.0092
	Vc·f	0.04836	1	0.04836	16.55448	0.0023
	Vc·d	0.012012	1	0.012012	4.112048	0.0701
	f·d	0.129541	1	0.129541	44.34354	<0.0001
	Residual	0.029213	10	0.002921	-	-
	R-Squared = 0.9977, Adj R-Squared = 0.9957 and Pred R-Squared = 0.9851					

**Table 7:** ANOVA table for micro-hardness.

Micro-Hardness	Source	Sum of Squares	Degree of Freedom	Mean Square	F-Value	*p*-Value
For Dry turning	Model	376.3987	9	41.82207	275.2646	<0.0001
	Vc	196.249	1	196.249	1291.672	<0.0001
	f	145.161	1	145.161	955.4209	<0.0001
	d	14.884	1	14.884	97.96353	<0.0001
	Vc^2^	7.652784	1	7.652784	50.3691	<0.0001
	f^2^	1.622784	1	1.622784	10.68084	0.0085
	d^2^	0.638409	1	0.638409	4.201882	0.0675
	Vc·f	0.10125	1	0.10125	0.666407	0.4333
	Vc·d	0.28125	1	0.28125	1.851132	0.2035
	f·d	1.05125	1	1.05125	6.919119	0.0251
	Residual	1.519341	10	0.151934	-	-
	R-Squared = 0.9960, Adj R-Squared = 0.9924 and Pred R-Squared = 0.9263					
For Cryogenic turning	Model	343.6334	9	38.18149	92.40286	<0.0001
	Vc	33.856	1	33.856	81.93476	<0.0001
	f	258.064	1	258.064	624.5395	<0.0001
	d	1.369	1	1.369	3.313111	0.0987
	Vc^2^	9.458182	1	9.458182	22.8897	0.0007
	f^2^	0.845682	1	0.845682	2.046631	0.1830
	d^2^	39.61506	1	39.61506	95.87222	<0.0001
	Vc·f	8.20125	1	8.20125	19.84781	0.0012
	Vc·d	0.01125	1	0.01125	0.027226	0.8722
	f·d	0.06125	1	0.06125	0.148231	0.7083
	Residual	4.132068	10	0.413207	-	-
	R-Squared = 0.9881, Adj R-Squared = 0.9774 and Pred R-Squared = 0.9192					

**Table 8:** Maximum percentage error between predicted and experimental values for the models developed for the surface roughness and micro-hardness of the AZ31C magnesium alloy.

Models	Maximum Percentage Error	
	Dry Turning	Cryogenic Turning
For Ra	0.298%	0.409%
For Rz	0.316%	0.724%
For Rt	0.557%	1.591%
For μH	0.756%	1.634%

**Table 9:** Optimization objectives and parameters, and their respective limits.

Name	Goal	Lower Limit	Upper Limit	Lower Weight	Upper Weight	Importance
Cutting velocity	is in range	50	150	1	1	3
Feed rate	is in range	0.1	0.14	1	1	3
Depth of cut	is in range	0.2	0.6	1	1	3
Ra, Cry	minimize	0.281	0.643	1	1	3
Rz, Cry	minimize	1.513	3.46	1	1	3
Rt, Cry	minimize	2.967	6.674	1	1	3
μH, Cry	maximize	82	97.2	1	1	3
MMR, Cry	maximize	1	12.6	1	1	3

**Table 10:** Optimal combination of process parameters with their respective responses.

Process Parameters/Responses	Optimal Solution	
	Case 1	Case 2
Vc (m/min)	150	150
f (mm/rev)	0.11	0.11
d (mm)	0.2	0.6
Ra (μm)	0.292	0.332
Rz (μm)	1.707	2.153
Rt (μm)	3.065	3.793
μH (HV)	90.79	90.8
MMR (mm^3^/min)	3.3	9.9
Desirability	0.837	0.71

## Data Availability

Not Applicable.
